# Cytarabine-Resistant *FLT3*-ITD Leukemia Cells are Associated with *TP53* Mutation and Multiple Pathway Alterations—Possible Therapeutic Efficacy of Cabozantinib

**DOI:** 10.3390/ijms20051230

**Published:** 2019-03-11

**Authors:** Ya-Chen Ko, Chung-Yi Hu, Zheng-Hau Liu, Hwei-Fang Tien, Da-Liang Ou, Hsiung-Fei Chien, Liang-In Lin

**Affiliations:** 1 Department of Medical Imaging and Radiological Technology, Yuanpei University of Medical Technology, Hsinchu 300, Taiwan; koyc@mail.ypu.edu.tw; 2 Department of Clinical Laboratory Sciences and Medical Biotechnology, College of Medicine, National Taiwan University, Taipei 100, Taiwan; jcyhu@ntu.edu.tw (C.-Y.H.); B99404035@ntu.edu.tw (Z.-H.L.); 3 Department of Laboratory Medicine, National Taiwan University Hospital, Taipei 100, Taiwan; 4 Internal Medicine, National Taiwan University Hospital, Taipei 100, Taiwan; hftien@ntu.edu.tw; 5 Department of Oncology, College of Medicine, National Taiwan University, Taipei 100, Taiwan; dlou@ntu.edu.tw; 6 Division of Plastic Surgery, Department of Surgery, Taipei Medical University Hospital, Taipei 110, Taiwan; 7 TMU Center for Cell Therapy and Regeneration Medicine, Taipei Medical University, Taipei 110, Taiwan

**Keywords:** cytarabine, acute myeloid leukemia, drug-resistance, *FLT3*-ITD

## Abstract

Internal tandem duplication of *FLT3* juxtamembrane domain (*FLT3*-ITD)-positive acute myeloid leukemia (AML) leads to poor clinical outcomes after chemotherapy. We aimed to establish a cytarabine-resistant line from *FLT3*-ITD-positive MV4-11 (MV4-11-P) cells and examine the development of resistance. The *FLT3*-ITD mutation was retained in MV4-11-R; however, the protein was underglycosylated and less phosphorylated in these cells. Moreover, the phosphorylation of ERK1/2, Akt, MEK1/2 and p53 increased in MV4-11-R. The levels of Mcl-1 and p53 proteins were also elevated in MV4-11-R. A p53 D281G mutant emerged in MV4-11-R, in addition to the pre-existing R248W mutation. MV4-11-P and MV4-11-R showed similar sensitivity to cabozantinib, sorafenib, and MK2206, whereas MV4-11-R showed resistance to CI-1040 and idarubicin. MV4-11-R resistance may be associated with inhibition of Akt phosphorylation, but not ERK phosphorylation, after exposure to these drugs. The multi-kinase inhibitor cabozantinib inhibited *FLT3*-ITD signaling in MV4-11-R cells and MV4-11-R-derived tumors in mice. Cabozantinib effectively inhibited tumor growth and prolonged survival time in mice bearing MV4-11-R-derived tumors. Together, our findings suggest that Mcl-1 and Akt phosphorylation are potential therapeutic targets for p53 mutants and that cabozantinib is an effective treatment in cytarabine-resistant *FLT3*-ITD-positive AML.

## 1. Introduction

Acute myeloid leukemia (AML) is a disorder of clonal hyperproliferation of abnormal myeloid progenitor cells emerging from a heterogeneous genetic background. Standard induction therapy for patients with AML consists of cytarabine and anthracycline (idarubicin or daunorubicin) treatment. After achieving complete remission, patients are given a consolidation therapy involving a high dose of cytarabine. Among the 50–75% of those who achieve complete remission after induction therapy, 80% suffer disease relapse attributed to drug resistance [1,2]. Hence, chemoresistance remains a major challenge in AML therapy.

Multiple mechanisms have been shown to relate to chemoresistance in AML. Cytarabine resistance in AML cells was shown to be linked to aberrant expression of equilibrative nucleoside transporters (ENT1) and metabolic enzymes deoxycytidine kinase (DCK) and cytosolic 5′-nucleotidase-II (NT5C2) [3,4,5]. Enhanced drug export activities of multidrug resistance proteins were also reported to be linked to AML chemoresistance [6,7]. Alternatively, leukemic cells may acquire chemoresistance by dysregulation of apoptosis regulators such as p53, Bcl-2, Bcl-xL, and Mcl-1 [8]. However, results for apoptosis regulation vary and are cell-type dependent [8,9,10,11]. Furthermore, the sterile alpha motif and histidine-aspartate domain-containing protein 1 (SAMHD1) was shown to hydrolyze the active metabolite of cytarabine, cytarabine triphosphate (ara-CTP), and also be involved in cytarabine resistance of AML cells [12,13].

FMS-like tyrosine kinase 3 internal tandem duplication (*FLT3*-ITD) is one of the most frequently found mutations in AML and is detected in 20–30% of adults with AML and in 10–15% of pediatric AML cases [14,15]. *FLT3*-ITD-positive patients are categorized in the poor prognostic group with a complete remission (CR) rate of 50–80% and a relapse rate of 70–80% with induction therapy without hematopoietic cell transplantation [2]. The affected FLT3 is a class III receptor tyrosine kinase highly expressed in hematopoietic stem and progenitor cells, and *FLT3*-ITD mutation leads to ligand-independent activation of downstream pathways [16]. Compared to AML cells with wildtype *FLT3*, AML cells with *FLT3*-ITD are linked to higher *Mcl-1* and lower *ENT1* expression, which may lead to reduced apoptosis and lower sensitivity to cytarabine in the mutant cells [17,18]. However, no study analyzed the effects of prolonged cytarabine exposure and the development of drug resistance in AML cells with *FLT3*-ITD. In the present study, we used the *FLT3*-ITD-positive AML cell line MV4-11 to generate the cytarabine-resistant cell line MV4-11-R. We analyzed the differences between the parental and the resistant cell lines in detail and evaluated the inhibition of MV4-11-R tumorigenicity by the multi-kinase inhibitor cabozantinib.

## 2. Results

### 2.1. Cytotoxicity Analyses, Growth Assessments, Morphology, and Surface Marker Expression of MV4-11-R

Cells were assessed for cytarabine sensitivity after establishment of the MV4-11-R cell line. Cytotoxicity analyses revealed that the IC_50_ values of cytarabine were 0.26 μM for MV4-11-P and 3.37 μM for MV4-11-R (Supplementary Figure S1A). Growth curves, cell cycle distribution, and apoptotic ratios showed minor differences between MV4-11-P and MV4-11-R (Supplementary Figure S1B–D). Cell morphology assessed by Liu’s staining showed no significant morphological changes (Supplementary Figure S2). Analysis of surface marker expression by flow cytometry showed no gross changes in the profile of CD markers, except an increase in CD56 (22.6% to 37.0%) and CD16 (5.7% to 8.8%) as well as a decrease in HLA-DR (12.7% to 2.4%) in MV4-11-R compared to MV4-11-P. Further analysis of expression levels of stem cell markers CD34 and CD117 showed no significant difference between MV4-11-P and MV4-11-R (Supplementary Table S1). Cytogenetic analyses revealed that clonal abnormalities t(4;11)(q21;q23), +8, dic(18;19)(p11;q11), +19 were present in both MV4-11-P and MV4-11-R. Expression of the *MLL-AF4* rearranged gene resulting from t(4;11) remained the same in both MV4-11-P and MV4-11-R. After establishing the MV4-11-R line, it was maintained in normal culture medium without further cytarabine treatment. Cell proliferation curves and cytarabine cytotoxicities were assessed over a two-year period; the phenotypes and the IC_50_ values of cytarabine of the resistant cell line remained stable over time.

### 2.2. FLT3-ITD Mutation and Activation Status in MV4-11-R

**Figure 1:**
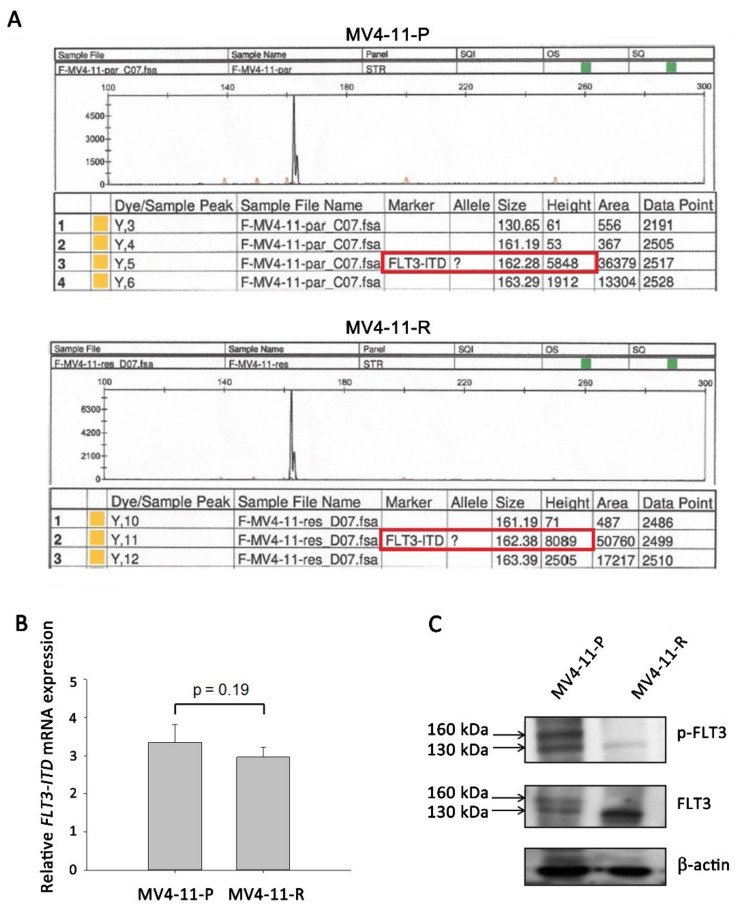
FMS-like tyrosine kinase 3 internal tandem duplication (*FLT3*-ITD) mutation and protein expression were detected in MV4-11-P and MV4-11-R cells. (**A**) GeneScan analysis showed the presence of the *FLT3*-ITD mutation (162bps, red frame) instead of wildtype FLT3 (130bps) in both MV4-11-P and MV4-11-R cells. (**B**) *FLT3*-ITD mRNA expression was revealed by qPCR. Data are representative of two independent experiments each performed in triplicate. (**C**) Total and phosphorylated FLT3 protein was observed in mature (160 kDa) and immature (130 kDa) forms. Representative Western blots of three independent experiments are shown.

MV4-11 possesses a homozygous *FLT3*-ITD mutation and clearly expresses the activated FLT3 receptor [19]. Using GeneScan analysis, the *FLT3*-ITD mutation was detected in both MV4-11-P and MV4-11-R (Figure 1A) without wildtype *FLT3*; no difference in *FLT3*-ITD mRNA expression between MV4-11-P and MV4-11-R was observed by qPCR (Figure 1B). However, Western blot analysis revealed that the FLT3 protein in MV4-11-R presents mainly in the immature (130 kD, underglycosylated) form rather than both the mature (160 kD, completely glycosylated) and immature forms detected in MV4-11-P (Figure 1C). 

**Figure 2:**
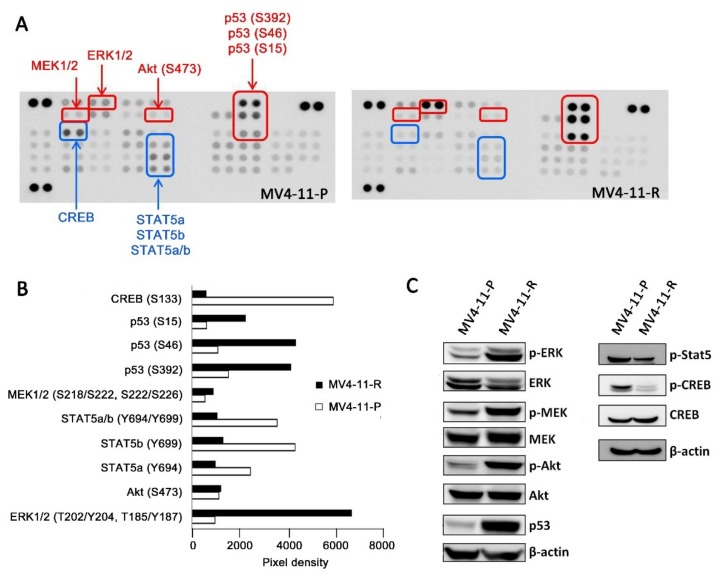
Differential kinase phosphorylation was observed between MV4-11-P and MV4-11-R cells. (**A**) Human phospho-kinase array blots showed increased (shown in red) and decreased (shown in blue) phosphorylation of multiple kinases in MV4-11-R compared with MV4-11-P. Spots for each phospho-kinase antibody are in duplicate. (**B**) Average pixel densities of duplicate spots on the human phospho-kinase array blots were analyzed using ImageJ. (**C**) Cell lysates of MV4-11-P and MV4-11-R were analyzed for selected total/phosphorylated kinases. Representative Western blots of three independent experiments are shown.

*FLT3*-ITD-positive cells exhibit ligand-independent autophosphorylation of the FLT3 receptor and activation of the downstream signaling cascade, including ERK, Akt, CREB, and STAT5 phosphorylation [16]. We demonstrated that, in contrast to MV4-11-P, the FLT3 receptor was less phosphorylated in MV4-11-R (Figure 1C). Antibody array analysis for human phospho-kinases showed that, in MV4-11-R, phosphorylation of certain residues on ERK1/2, Akt, MEK1/2, and p53 increased; on the other hand, phosphorylation of STAT5a, STAT5b, and CREB decreased (Figure 2A, B). We further verified the increased phosphorylation of ERK1/2 (Thr202/Tyr204), MEK1/2 (Ser218/Ser222), Akt (Ser473), and total p53 as well as the decreased phosphorylation of STAT5 (Tyr694) and CREB (Ser133) by Western blot analyses (Figure 2C). In addition, we observed significantly increased amount of p53 protein in MV4-11-R relative to that in MV4-11-P (Figure 2C).

### 2.3. Apoptosis-Related Proteins in MV4-11-R

**Figure 3:**
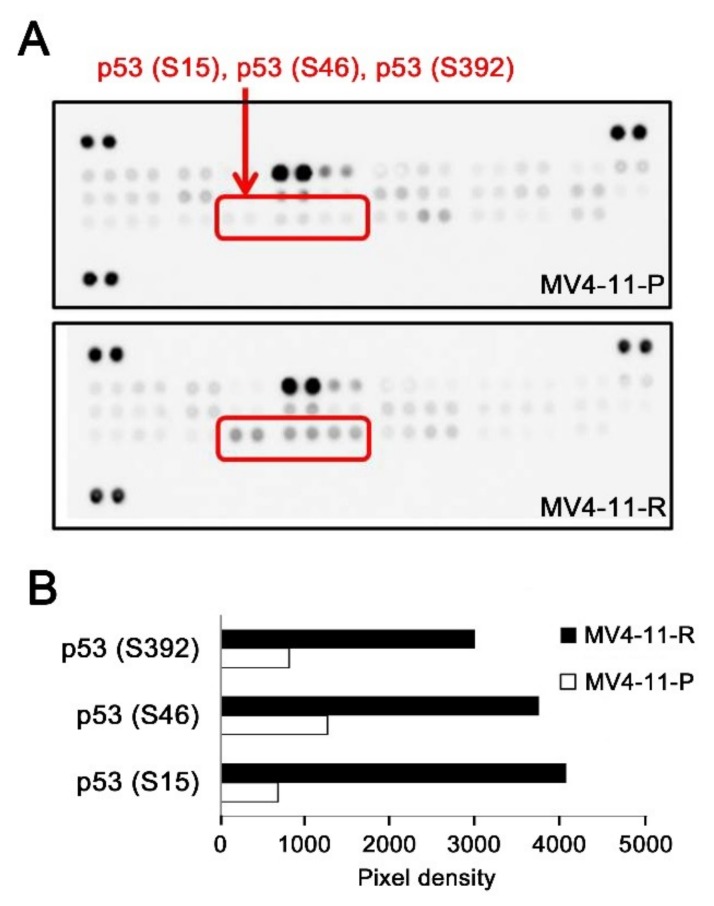
Apoptosis-related proteins in MV4-11-P and MV4-11-R were analyzed by human apoptosis array. (**A**) Blots of the array show increased p53 phosphorylation at serine 15, 46, and 392 in MV4-11-R compared to MV4-11-P. (**B**) Average pixel densities for duplicate spots of phosphorylated p53 were analyzed using ImageJ.

Since leukemic cells can acquire chemoresistance by abolishing sensitivity to apoptosis, we screened for apoptosis-related protein expression in MV4-11-P and MV4-11-R. Using the antibody array, we observed that p53 phosphorylation at Ser15, Ser46, and Ser392 was higher in MV4-11-R compared to MV4-11-P (Figure 3), which also confirmed our previous data in Figure 2. 

**Figure 4:**
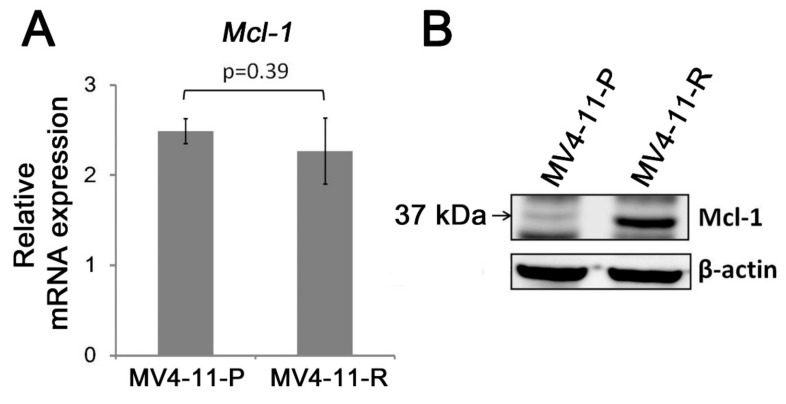
Differential Mcl-1 protein expression was observed between MV4-11-P and MV4-11-R. (**A**) *Mcl-1* mRNA levels show no significant difference between MV4-11-P and MV4-11-R by qPCR. Quantitative data are representative of three independent experiments. (**B**) Western blot analysis shows that Mcl-1 protein expression is increased in MV4-11-R. Representative Western blots from three independent experiments are shown.

Mcl-1 is an anti-apoptotic protein of the Bcl-2 family and is highly expressed in hematopoietic stem cells and leukemic stem cells. In *FLT3*-ITD-positive AML cells, Mcl-1 expression is upregulated in leukemic stem cells by *FLT3*-ITD-specific STAT5 activation [19]. Our data showed that Mcl-1 was substantially increased at the protein level (Figure 4B). However, no significant change was observed at the *Mcl-1* mRNA level (Figure 4A).

### 2.4. An Additional TP53 Mutation Emerged in MV4-11-R

**Figure 5:**
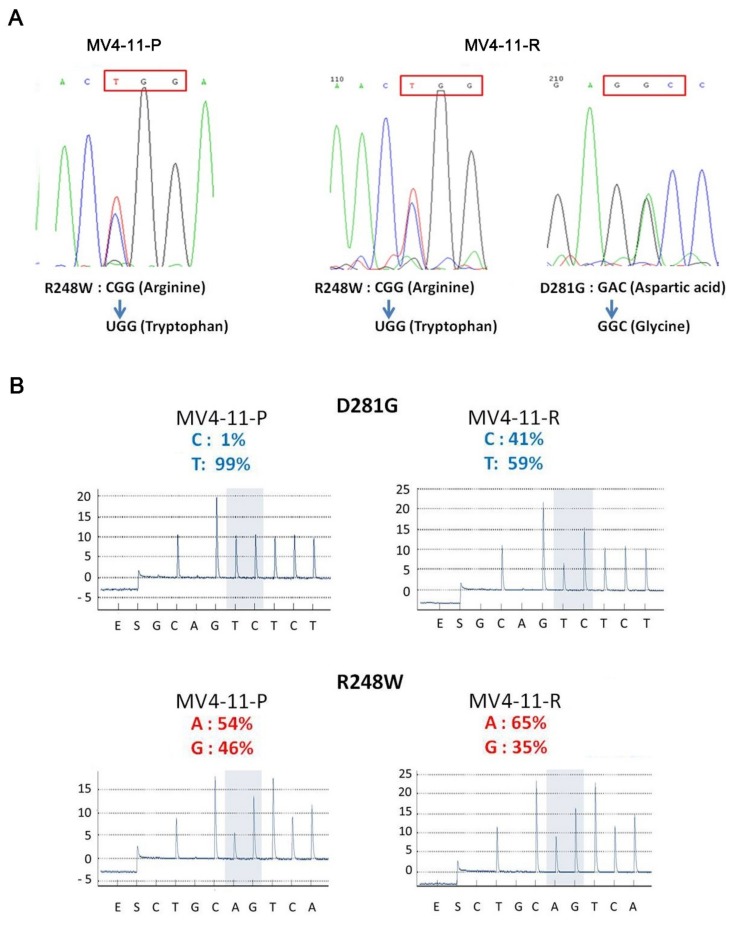
Sequencing analyses of the *TP53* gene reveal the emergence of a new *TP53* mutation, D281G, in MV4-11-R. (**A**) The R248W (CGG → TGG, red frame) *TP53* mutation was detected in MV4-11-P, while both R248W and D281G (GAC → GGC, red frame) mutations were observed in MV4-11-R using Sanger sequencing analysis. (**B**) The percentage of mutant antisense-alleles for D281G and R248W mutations in MV4-11-P and MV4-11-R was determined by pyrosequencing.

The wild-type p53 protein functions as a tumor suppressor to promote cell senescence and trigger apoptosis; however, we observed higher amounts of p53 protein in MV4-11-R. Mutations in the *TP53* gene were shown to correlate with the growth-inhibitory potency of chemotherapeutic drugs in a number of cancer cell lines, including leukemia cell lines [20,21]. We analyzed the *TP53* gene sequence in MV4-11-P, showing that it is mutated at codon 248 from CGG (arginine) to UGG (tryptophan), designated as the R248W mutation. In MV4-11-R, we detected another point mutation at codon 281 from GAC (aspartic acid) to GGC (glycine), designated as the D281G mutation (Figure 5A), in addition to the R248W mutation. Pyrosequencing analysis revealed that the percentage of D281G mutant alleles increased from 1% to 41% during the transition of MV4-11-P to MV4-11-R, while the percentage of R248W mutant alleles only slightly shifted from 54% to 65% (Figure 5B). Further cloning analysis verified that most D281G alleles were from wildtype R248 alleles, resulting in only 13.3% wild-type *TP53* alleles remained in MV4-11-R cells against 43.5% wild-type *TP53* alleles in MV4-11-P cells. This suggests that a cell population harboring the D281G mutation emerged in the MV4-11-R line, and the reduction in wild-type p53 resulted in a growth advantage compared to MV4-11-P cells.

To answer whether *TP53* mutations associate with cytarabine resistance, we compared *TP53* status among cell lines from the National Cancer institute-60 (NCI-60) panel and their IC_50_ data for cytarabine from online database CancerDR [22,23]. It showed that cell lines bearing *TP53* mutations tend to have higher IC_50_ of cytarabine (Supplementary Figure S3, Supplementary Table S2). Using data from Genomics of Drug Sensitivity in Cancer [24], a possible link was observed between *TP53* mutations and increased cytarabine resistance from data of 876 cancer cell lines (*p* = 0.0321), although it is not defined as a significant correlation due to high false discovery rate (FDR%) (Supplementary Table S3). These data further support that the emergence of a *TP53* mutation in MV4-11-R may contribute to cytarabine resistance.

### 2.5. Examination of the Cytarabine Metabolic Pathway and Multidrug Resistance Genes in MV4-11-R 

We assessed whether transporters and enzymes in the cytarabine metabolic pathway are involved in cytarabine resistance in MV4-11-R. Our qPCR results showed that there are no significant differences in the mRNA expression of *DCK*, *SAMHD1*, *NT5C2*, and *ENT1* between MV4-11-P and MV4-11-R. We also examined the expression of ATP-binding cassette transporters such as multidrug resistance 1 (*MDR1*) and multidrug resistance-associated protein 1 (*MRP1*), which were linked to chemoresistance in tumor cells. No significant difference was revealed in the mRNA expression of *MDR1* and *MRP1* between MV4-11-P and MV4-11-R. 

### 2.6. Cabozantinib Effectively Inhibits Tumorigenic Features of MV4-11-P and MV4-11-R Both In Vitro and In Vivo

**Figure 6:**
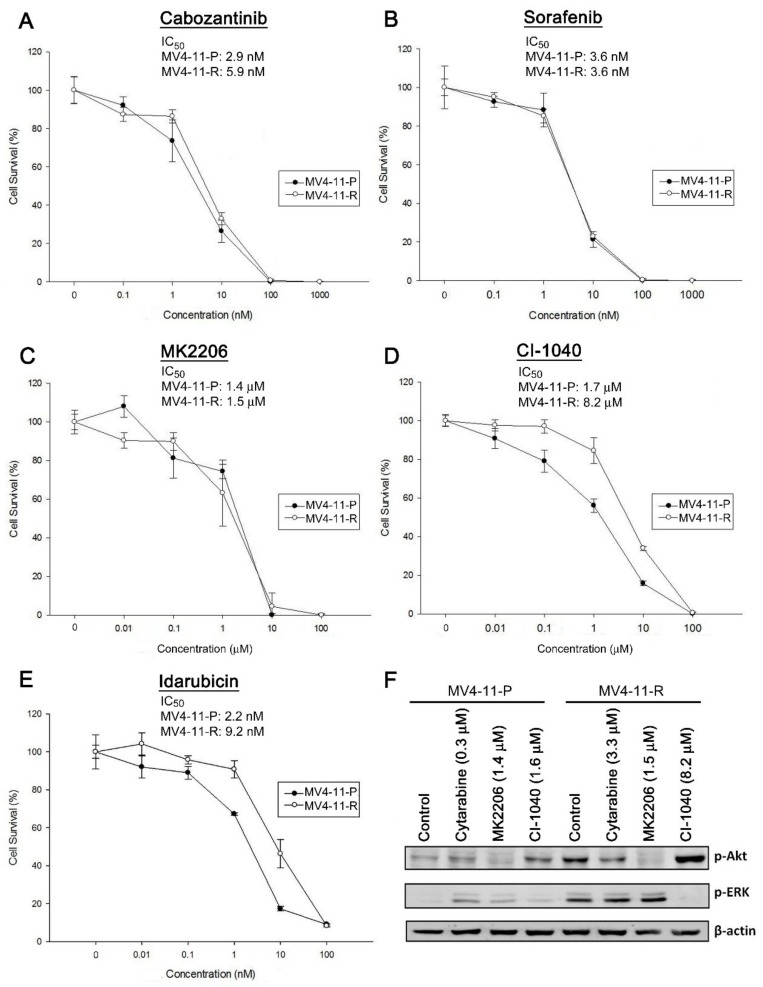
(**A**–**E**) Cytotoxicity curves and IC_50_ values of anti-cancer drugs for MV4-11-P and MV4-11-R were evaluated by MTS assays. Data are representative of at least two independent experiments each performed in triplicate. Cells were drug-treated for 72 h before performing the MTS assay. (**F**) Phosphorylation of Akt and ERK in response to drug treatment in MV4-11-P and MV4-11-R was detected by Western blot analysis. Cells were treated with cytarabine, MK2206, or CI-1040 for 6 h before collection of cell lysates. DMSO (0.01%) served as a control. Representative Western blots of two independent experiments with similar results were shown.

**Figure 7:**
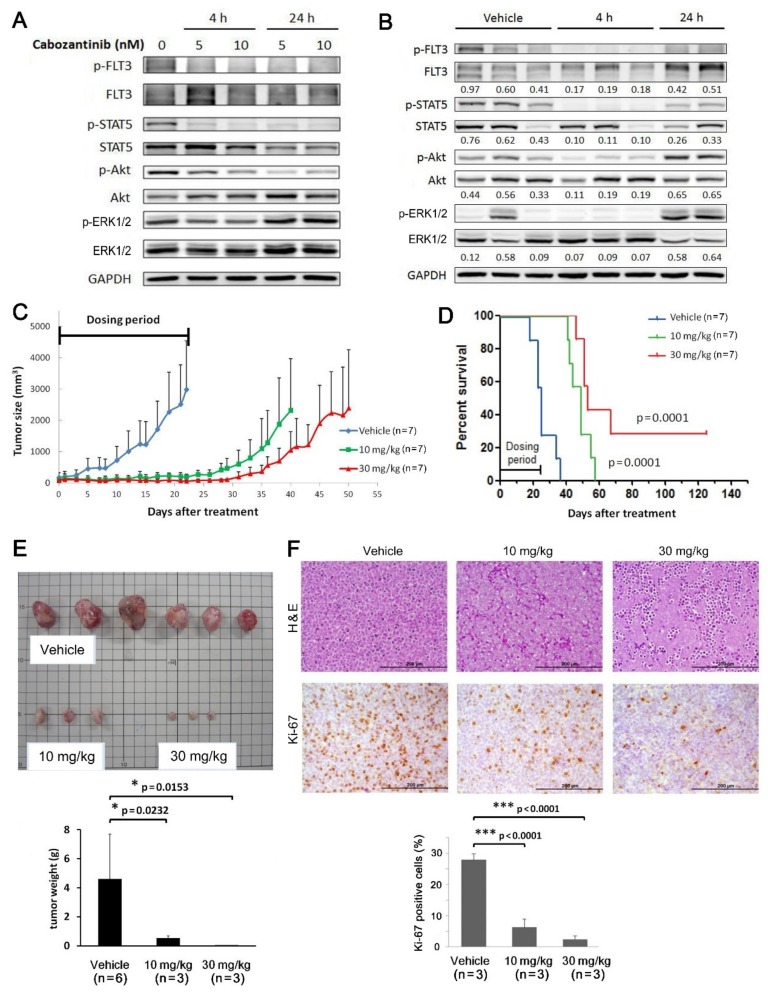
Cabozantinib inhibits *FLT3*-ITD signaling in MV4-11-R cells and growth of MV4-11-R-derived tumors. Kinase phosphorylation of the *FLT3*-ITD signaling pathway in cultured MV4-11-R cells was evaluated by Western blot analysis (**A**). MV4-11-R tumor xenografts were grown in nude mice; when tumor sizes reached 100–200 mm^3^, mice were then given 30 mg/kg of cabozantinib-malate in a single dose. Mice were sacrificed after 4 h or 24 h, and protein extracts were prepared from the tumor tissues and analyzed by Western blotting (**B**). To assess tumor growth, mice with MV4-11-R xenograft tumors reaching 100–200 mm^3^ were randomly given vehicle, 10 mg/kg, or 30 mg/kg cabozantinib-malate daily in four five-day courses (days 1–5, 7–11, 13–17, and 19–23). Tumor volumes were measured every 1–2 days (**C**), and Kaplan–Meier survival curves of the different mice groups were plotted against the number of days after treatment (**D**). Multiple tumors from each treatment group were obtained, weighed (**E**), stained with hematoxylin and eosin (H&E) or Ki-67 antibodies, and quantified for the number of Ki-67-positive cells (**F**). Quantification data represents the mean ± SD from three independent experiments. *, *p* < 0.05; **, *p* < 0.01; ***, *p* < 0.001. Scale bars = 200 μM.

We further tested the responses of MV4-11-P and MV4-11-R to a number of anti-cancer drugs. MV4-11-P and MV4-11-R cells showed similar sensitivity to cabozantinib (a multi-kinase inhibitor), sorafenib (a multi-kinase inhibitor), and MK2206 (an Akt inhibitor) (Figure 6A–C). On the other hand, MV4-11-R was less sensitive than MV4-11-P to CI-1040 (a MEK inhibitor) or idarubicin; the IC_50_ values for both drugs in MV4-11-R were approximately five-fold higher than those in MV4-11-P (Figure 6D,E). Further examination of ERK and Akt phosphorylation showed that elevated Akt phosphorylation in MV4-11-R was effectively inhibited by cabozantinib and MK2206, but not by CI-1040 (Figure 6F and Figure 7A); elevated ERK phosphorylation in MV4-11-R was inhibited effectively by CI-1040 (Figure 6F), but not by MK2206 (Figure 6F) and cabozantinib (Figure 7A). Taken together, the drug clearance efficacy of MV4-11-R seems to correlate with its ability to inhibit phosphorylation of Akt but not ERK. In addition, *TP53* mutations also showed a possible correlation with increased resistance to CI-1040 (*p* = 0.00882) in cancer cell lines from the Genomics of Drug Sensitivity in Cancer database (Supplementary Table S2) [24]. No correlation was observed between *TP53* mutations and resistance to cabozantinib, sorafenib, or MK2206. This evidence is in line with our results on drug sensitivity in MV4-11-P and MV4-11-R (Figure 6A–D).

Among the drugs similarly effective for MV4-11-P and MV4-11-R, we chose to focus on examining the therapeutic potential of cabozantinib towards MV4-11-R because of our previously reported effect of cabozantinib towards MV4-11-P [25]. In this study, cabozantinib effectively inhibited *FLT3*-ITD-dependent signaling in MV4-11-R cells, including phosphorylation of FLT3, STAT5, and Akt; however, ERK phosphorylation was only transiently and weakly inhibited (Figure 7A). We also observed a similar inhibition of FLT3, STAT5, and Akt phosphorylation by oral treatment with cabozantinib in vivo through analyzing protein extracts from MV4-11-R-derived tumors in nude mice (Figure 7B). As shown in our previous report in MV4-11-P cells [25], cabozantinib also effectively inhibited the growth of an MV4-11-R-derived tumor in mice during the dosing period (Figure 7C). Survival time was significantly longer in the cabozantinib-treated groups compared to that in the vehicle-treated group (Figure 7D). Average tumor weight was lower in the cabozantinib-treated group (Figure 7E). Additionally, staining of tumor sections with a Ki-67 antibody revealed significantly lower cell proliferation in tumor tissues from cabozantinib-treated mice than those in vehicle-treated mice (Figure 7F).

## 3. Discussion

Patients with AML often relapse after chemotherapy, and *FLT3*-ITD is one of the most frequently found mutations in AML patients. In the present study, we successfully established a cytarabine-resistant cell line, called MV4-11-R, from the parental cell line MV4-11-P and characterized its growth properties, phosphorylation of kinases, *FLT3*-ITD mutation status and signaling, *TP53* expression and mutations, cytarabine metabolic enzymes and transporters, and responses to anti-cancer drugs (especially cabozantinib) both in vitro and in vivo. 

For MV4-11-R, the IC_50_ of cytarabine was 3.37 μM, which is approximately 13-fold higher than that for MV4-11-P (0.26 μM). Previous studies examining the plasma level during cytarabine treatment showed that plasma concentrations of 5–40 μM were reached during high-dose (3 g/m^2^) cytarabine infusion [26]. Intermediate-dose (500 mg/m^2^) treatment yielded 10–15 μM steady-state plasma concentrations [27,28]; in contrast, conventional-dose (50–200 mg/m^2^) treatments generated steady-state plasma concentrations between 0.1 μM and 1.0 μM [26,27,28]. The IC_50_ of MV4-11-R corresponds to the plasma concentration in patients receiving between conventional and intermediate-dose infusion. Since 3.37 μM is the IC_50_ value obtained for a 72-hour culture, a considerably higher concentration of cytarabine is required to completely eliminate MV4-11-R. Therefore, MV4-11-R can be evaluated as a cytarabine-resistant cell line suitable for assessment of changes in the development of drug resistance. MV4-11 cell line expresses an *MLL-AF4* rearranged gene resulted from the t(4;11) translocation. *MLL* gene-rearranged leukemia was reported to be more sensitive to cytarabine treatment [29,30]. In addition, MV4-11-P and MV4-11-R showed similar *MLL-AF4* expression. Therefore, *MLL*-rearrangement would hardly be considered a driving force for cytarabine resistance.

We demonstrated that the FLT3 protein exists in an immature, underglycosylated form in MV4-11-R. Glycosylation of the FLT3 receptor starts in the endoplasmic reticulum (ER) and is completed in the Golgi apparatus, from where it is delivered to the cell membrane. It was reported that the constitutive kinase activity of the FLT3 protein is linked to its inefficient folding and ER retention [31], leading to a decreased amount of receptors on the cell surface. Our findings suggest that cells predominantly bearing immature FLT3 were enriched in MV4-11-R, showing a possible growth advantage over cells with higher levels of mature FLT3. Immature, ER-bound FLT3 in MV4-11-R may result in weaker FLT3 phosphorylation and less STAT5 activation as we have observed. Therefore, the strong phosphorylation of ERK and Akt observed in MV4-11-R is responsible for its growth advantage and can result from the aberrant activation of other signaling pathways. We assume that MV4-11-P cells with high FLT3 phosphorylation undergo *FLT3*-ITD-dependent growth and are therefore more sensitive to cytarabine, whereas a fraction of cells with ER-bound and less phosphorylated FLT3 exhibits *FLT3*-ITD-independent growth and develops cytarabine resistance. Therefore, targeted inhibition of ERK and Akt can be a promising strategy to overcome cytarabine resistance in AML.

Our data show that the anti-apoptotic Mcl-1 is increased at the protein level but not at the transcription level in MV4-11-R. The Mcl-1 protein has a short half-life and its stability can be controlled by post-translational regulation such as ubiquitination [32,33,34]. It was demonstrated that Mcl-1, but not other Bcl-2 family proteins (such as Bcl-xL, Bcl-2, or Bcl-w), is essential for maintaining AML cell growth both in vitro and in vivo [35], indicating the importance of Mcl-1 in AML. Mcl-1 upregulation is also responsible for *FLT3*-ITD-mediated drug resistance of cytotoxic agents including cytarabine [36]. A further increase in Mcl-1 proteins in MV4-11-R suggested that cytarabine resistance can arise from a subpopulation of cells having elevated Mcl-1 expression. Our data support the notion that a combination of anti-Mcl-1 drugs with chemotherapy is a promising therapeutic strategy for *FLT3*-ITD-positive AML [37,38].

We observed the emergence of a D281G p53 mutant in MV4-11-R, in addition to the original R248W mutation in MV4-11-P. *TP53* mutations were reported to associate with poor prognosis in de novo AML [39]. D281G and R248W mutations are both located in the DNA-binding domain of p53 and are considered gain-of-function mutations [40]. Previous studies showed that both D281G and R248W mutants transactivate expression of tumor progression-related genes such as *MYC*, *PCNA*, *EGFR*, and *EGR1*; the D281G mutant shows an even greater transactivating activity than R248W in these studies [41,42,43,44]. As a result, cells carrying a D281G mutation in MV4-11-R exert a survival advantage against those carrying only R248W and were selected for cytarabine resistance. Therefore, a screen of possible p53 mutations should be considered, and p53 mutants can be targeted for therapy of chemoresistant *FLT3*-ITD-positive AML.

Our results showed that p53 phosphorylation at Ser15, Ser46, and Ser392 was increased in MV4-11-R. We reason that the increase in p53 phosphorylation is due to a higher amount of total p53 protein expressed in MV4-11-R. The Ser15 and Ser46 phosphorylation sites are both located in the N-terminal transactivation domains of p53. Although phosphorylation at these two sites enhances apoptosis triggered by wild-type p53 [45], Ser15 phosphorylation enhances the stability of mutant p53 [46,47]. Ser392 is located in the C-terminal regulatory domain, and hyperphosphorylation at Ser392 was observed in tumor samples and cell lines [48]. Phosphorylation at Ser392 stabilizes the gain-of-function mutant p53 tetramer and potentiates its oncogenicity [49]. Our results are mostly in line with these observations and suggest that increased phosphorylation at Ser15, Ser46, and Ser392 of mutant p53 provides a further growth advantage to MV4-11-R cells by stabilizing the mutant p53 protein.

Prolonged drug exposure can result in selective survival in favor of certain pre-existing subclones within the heterogeneous cancer cell pool. It can also induce certain events including mutations, epigenetic changes, or non-genetic transcriptional variability in a limited number of cells [50]. Our pyrosequencing data show that a new D281G mutation exists in MV4-11-R (41%) (Figure 5B). This mutation was also detected in MV4-11-P at a trace amount (1%); therefore, we consider MV4-11-R to be developed from certain subclones selected by cytotoxic pressure from cytarabine.

Exposure to chemotherapy agents may lead to the development of therapy-related AML (t-AML) in cancer patients. *TP53* was the most frequently mutated gene found in t-AML. An existing *TP53* E286K mutant clone (2.5%) was identified at first relapse and expanded to more than 30% at second relapse, compatible with t-AML [39]. Another study showed that *TP53* mutations were significantly enriched in t-AML compared with de novo AML. These *TP53* mutations were presented at low frequency before any chemotherapy or years before the diagnosis of t-AML [51]. These clinical findings suggested that the increased percentage of the D281G mutant in MV4-11-R is a clinically related event, and *TP53* mutation is an important therapeutic target for chemoresistance and t-AML.

In order to mimic cytarabine resistance evolved in AML patients, a number of studies were done using resistant AML cell lines established by prolonged cytarabine exposure. The resistant cell lines exhibit increased ERK and Akt activation compared to the parental cell lines [11,52], as shown in our study. The major mechanism of cytarabine resistance described in these cell lines is down-regulation or loss of activity of the DCK [5,53,54,55,56], the rate-limiting enzyme to convert cytarabine to its active form ara-CTP. Other findings include increased expression of c-Myc, increased 5′-nucleotidase cN-II/NT5C2 activity, and loss of wild-type p53 [11,52,55]. We did not observe a significant change in expression of *NT5C2* mRNA. In vitro data showed that SAMHD1 decreased cytarabine sensitivity through its hydrolyzing activity to ara-CTP [56]. Clinical data revealed that AML patients with lower *SAMHD1* expression correlated with better response to cytarabine therapy [12,56]. However, SAMHD1 acts also as a tumor suppressor through its activity as a dNTP triphosphohydrolase to diminish intracellular dNTP pool and inhibit DNA replication [13]. In the present study, we did not find significant difference of the *SAMHD1* expression between MV4-11-P and MV4-11-R cells, demonstrating SAMHD1 did not play important role in this resistance. 

Altered phenotypes were described in cytarabine-resistant cells derived from parental AML cells. It was shown that the resistant cells possess enhanced susceptibility to NK-mediated lysis [52] and increased sensitivity to glucocorticoids [57], while the latter is only observed in wild-type FLT3 AML patient samples. Cytarabine-resistant cells isolated from an in vivo system showed increased inflammatory response, reactive oxygen species (ROS) production, and mitochondrial oxidative respiration [58]. Cross-resistance to cytarabine was found in doxorubicin-resistant cells isolated from prolonged doxorubicin exposure [59]. The double-resistant cells displayed a higher amount of the ATP-binding cassette transporter P-glycoprotein encoded by the *MDR1* gene and loss of histone methyltransferase EZH2 [6,60,61]. We also observed cross-resistance of idarubicin in MV4-11-R cells. Our qPCR data showed no difference in *MDR1* and *MRP1* expression, and the two drugs are metabolized differently within the cells [62]. Mechanisms of cross-resistance can be further assessed by establishing and characterizing idarubicin-resistant cell lines from MV4-11.

Our data revealed that Akt and ERK phosphorylation are significantly elevated in MV4-11-R. We speculate that the effectiveness of the anti-cancer drug correlates with its ability to inhibit Akt phosphorylation in MV4-11-R. The decreased sensitivity of MV4-11-R to CI-1040, idarubicin, and cytarabine may result from the failure of these drugs to inhibit Akt phosphorylation. On the other hand, our data suggest that the ERK phosphorylation level does not correlate with the decreased sensitivity of MV4-11-R to these anti-cancer drugs. We hypothesize that strong Akt phosphorylation, but not ERK phosphorylation, results in cytarabine resistance in MV4-11-R. According to the IC_50_ data, we showed that cabozantinib, sorafenib, and MK2206 are as potent for MV4-11-R as for MV4-11-P. Using a mouse xenograft model, we demonstrated that cabozantinib effectively inhibits the *FLT3*-ITD pathway along with MV4-11-R-derived tumor growth. These observations strongly support the therapeutic potential of cabozantinib in combination with cytarabine to treat *FLT3*-ITD-positive AML. 

## 4. Materials and Methods

### 4.1. Cell Culture and Establishment of the Cytarabine-Resistant Cell Line

MV4-11 cells were kindly provided by Yen-Chun Chen of the Industrial Technology Research Institute, Hsinchu, Taiwan, and were cultured in RPMI 1640 media supplemented with 10% fetal bovine serum at 37 °C in 5% CO_2_. Cytarabine was purchased from the the Pharmacia & Upjohn Company LCC (Kalamazoo, MI, USA). To establish a cytarabine-resistant line, MV4-11 cells were grown in gradually increasing concentrations of cytarabine. Before each adjustment of cytarabine concentration, the growth status of cells was confirmed to reach the growth rate in the parental line using the trypan blue exclusion method. The established cell line that was named MV4-11-R and the parental line was termed MV4-11-P. Both MV4-11-P and MV4-11-R cells were authenticated with 16-marker short tandem repeat (STR) by the Food Industry Research and Development Institute, Hsinchu, Taiwan. The genetic profiles of these two cells were identical to the reported genetic profiles. 

### 4.2. Cytotoxicity Curves

MV4-11-P or MV4-11-R cells were seeded in 96-well plates at 2500 cells/well. After drug treatment for 72 h, cells were incubated with CellTiter 96^®^ Aqueous One solution (20 μL in each well) at 37 °C for 2 h. Absorbance at 490 nm for each well was obtained. For each experimental set, a standard curve representing the relationship between the absorbance and the actual viable cell number was done in parallel. Cell viability was calculated as a percentage of the control (cell number of experimental group/cell number of control group).

### 4.3. Cytogenetics

Chromosome analysis was performed as described previously [63]. Briefly, bone-marrow cells from AML patients were used immediately or after 1–3 days of non-stimulated culturing. Metaphase chromosomes were visualised by the conventional trypsin–Giemsa banding technique and were karyotyped according to the International System of Human Cytogenetic Nomenclature [63].

### 4.4. RNA Extraction, Reverse Transcription, and Real-Time Quantitative PCR (qPCR)

Cellular RNAs were extracted using TRIzol^®^ Reagent (Life Technologies, Carlsbad, CA, USA) followed by DNase I treatment. Reverse transcription was performed using High-Capacity cDNA Reverse Transcription kits (Applied Biosystems, Foster City, CA, USA) according to the manufacturer’s instructions. qPCR was performed using synthesized cDNA and SYBR Green PCR Master Mix (Applied Biosystems) with initial denaturation at 95 °C for 10 min followed by 40 thermal cycles comprising 95 °C for 15 s and 60 °C for 1 min in an ABI 7500 Fast Real-Time PCR system (Applied Biosystems). Dissociation curves were obtained after the qPCR cycles to detect melting temperatures of the products by descending from 95 °C to 60 °C. *HPRT* expression was analyzed as the internal control. Relative target gene expression was calculated as 2^–ΔCT^ where ΔC_T_ = C_T(target gene)_ − C_T(*HPRT*)_. The primers used for qPCR reactions are 5′-CCACCCCGCCCAAGAG-3′ (forward) and 5′-CCTTCCCTGCAGCGATGTTCCC-3′ (reverse) for *DCK*; 5′-CCCGTGAAGAGTTCCCTGTC-3′ (forward) and 5′-GTAGGGGGCTAGAAGGGTGA-3′ (reverse) for *SAMHD1*; 5′-TGCGCTGGAGCCGAATAC-3′ (forward) and 5′-CCCTACTTCCTTCATCCGGC-3′ (reverse) for *NT5C2*; 5′-TGCATCCTGAAAGCTGCGTA-3′ (forward) and 5′-GGCATTCTCTGGCTGTCACT-3′ (reverse) for *CDA*; 5′-GCAGCTGGAAGACAAATACACAAA-3′ (forward) and 5′-CCCCAACATCGTGCACATC-3′ (reverse) for *MDR1*, 5′-TGTTTCCAGCCGTGACT-3′ (forward) and 5′-CAGGCCACATGAATACAG-3′ (reverse) for *ENT1*; 5′-AGGTCAAGCTTTTCCGTGTACTG-3′ (forward) and 5′-GGACTTTCGTGTGCTCCTGA-3′ (reverse) for *MRP*; 5′-GCAATTTAGGTATGAAAGCCAGC-3′ (forward) and 5′-CTTTCAGCATTTTGACGGCAACC-3′ (reverse) for *FLT3*-ITD; 5′-AAGAGGCTGGGATGGGTTTGTG-3′ (forward) and 5′-TTGGTGGTGGTGGTGGTTGG-3′ (reverse) for *Mcl-1*; 5′-TGACACTGGCAAAACAATGCA-3′ (forward) and 5′-GGTCCTTTTCACCAGCAAGCT-3′ (reverse) for *HPRT*.

### 4.5. DNA Extraction, GeneScan Analysis, and DNA Sequencing

The QIAamp DNA Mini kit (QIAGEN, Hilden, Germany) was used for cellular DNA extraction according to the manufacturer’s instructions. For GeneScan analysis of the *FLT3*-ITD mutation, PCR fragments were amplified with fluorescence-labelled primers, diluted with distilled water, and mixed with deionized formamide and GeneScan Size Standard (Applied Biosystems). Mixtures were electrophoresed on an ABI 3100 Genetic Analyzer (Applied Biosystems), and fluorescence signals were analyzed by GeneScan, Volume 3.1 software (Applied Biosystems). For DNA sequencing of *TP53*, we purified PCR products amplified from primers 5′-GCCAAGTCTGTGACTTGCACG-3′ (forward) and 5′-TCAGTCTGAGTCAGGCCCTTCT-3′ (reverse), and then sequenced with the primers 5′-GCGTGTGGAGTATTTGGATG-3′ using the BigDye terminator v3.1 Cycle Sequencing kit (Applied Biosystems) and a 3730xl DNA Analyzer (Applied Biosystems).

### 4.6. Pyrosequencing

PCRs for pyrosequencing were performed using cDNA and the primers 5′-TTATCCGAGTGGAAGGAAATTTG-3′ (forward) and 5′-TCTTCTTTGGCTGGGGAGAGG-3′ (reverse). PCR products were immobilized on streptavidin-coated beads, denatured, and mixed with sequencing primers 5′-TGGTGAGGATGGGCC-3′ and 5′-TCTTCCTCTGTGCGC-3′ for R248W and D281G, respectively. Sequencing reactions were performed using the PyroMark Q24 instrument (QIAGEN) and analyzed by the PyroMark Q24 software (QIAGEN).

### 4.7. Western Blot Analysis

Cultured cells were lysed in lysis buffer containing 50 mM Tris-HCl pH 8.0, 150 mM NaCl, 1% NP-40, 0.5% sodium deoxycholate, 0.1% SDS, protease inhibitor cocktail (Roche Applied Science, Basel, Switzerland), and PhosStop^TM^ phosphatase inhibitor mixture (Roche Applied Science). Protein concentrations were determined using the Quick Start^TM^ Bradford Protein Assay (Bio-Rad Laboratories, Hercules, CA, USA). Lysates containing equal amounts of protein were subjected to SDS-PAGE and transferred to PVDF membranes (Millipore, Billerica, MA, USA). Target proteins on the membranes were detected with specific primary antibodies and matching horseradish peroxidase-conjugated secondary antibodies and visualized using Western Lightning^®^ Plus-ECL (Perkin Elmer, Waltham, MA, USA) and a LAS 4000 image reader (Fujifilm, Tokyo, Japan). The primary antibodies used were as follows: anti-FLT3, anti-MEK1/2, anti-p-MEK1/2, and anti-p53 antibodies (all from Santa Cruz Biotechnology, Santa Cruz, CA, USA); anti-p-FLT3, anti-ERK1/2, anti-p-ERK1/2, anti-Akt, anti-p-Akt, anti-p-STAT5, and anti-β-actin antibodies (Cell Signaling Technology, Beverly, MA, USA); anti-CREB and anti-p-CREB antibodies (Epitomics, Burlingame, CA, USA); anti-Mcl-1 and anti-GAPDH antibodies (GeneTex, San Antonio, TX, USA).

### 4.8. Analysis of Kinase Phosphorylation and Apoptosis-Related Protein Profiles

Cell lysates were analyzed using the Human Phospho-Kinase Array kit (R&D Systems, Minneapolis, MN, USA) and the Human Apoptosis Array kit (R&D Systems) according to the manufacturer’s instructions. Chemiluminescent signals were captured and analyzed by a LAS 4000 image reader (Fujifilm).

### 4.9. Mouse Xenograft Experiments

Female BALB/c athymic (nu+/nu+) mice (6–8 weeks old) were obtained from the National Laboratory Animal Center, Taipei, Taiwan. The animal protocols were approved by the Institutional Animal Care and Use Committee (IACUC) of National Taiwan University in accordance with the approved guidelines. The animal experimental protocol was modified from our previous report [25]. Each mouse was inoculated subcutaneously with 1 × 10^6^ MV4-11-R cells in 100 μL of 50% BD Matrigel^TM^ Matrix (BD Biosciences, San Jose, CA, USA). When the tumor size reached 200 mm^3^, mice were randomly grouped and orally administered cabozantinib-malate or vehicle by gavages. Body weights and tumor sizes were measured every 1–2 days after inoculation. When tumor volume reached 2000 mm^3^, animals were euthanized. Tumors were collected for Western blot analyses and immunohistochemical staining as described previously [25]. For immunohistochemical staining, tumor sections were labelled with an anti-Ki-67 antibody (Abcam, Cambridge, MA, USA).

### 4.10. Statistical Analysis

Quantitative data of mRNA expression, tumor sizes, and Ki-67-positive cells between experimental groups were analyzed by two-tailed Student’s *t*-tests. Survival of mice was analyzed by Kaplan–Meier survival curves and log-rank tests. *p* values less than 0.05 were considered statistically significant.

## 5. Conclusions

In this study, we have successfully established a cytarabine-resistant *FLT3*-ITD-positive cell line, MV4-11-R. A number of major alterations have been identified in MV4-11-R including decreased glycosylation and phosphorylation of the *FLT3*-ITD protein, increased ERK, Akt, and p53 phosphorylation, accumulation of the Mcl-1 protein, and emergence of the D281G gain-of-function p53 mutation. The multi-kinase inhibitor cabozantinib was shown to effectively inhibit the growth of cytarabine-resistant *FLT3*-ITD-positive AML cells in vivo. We propose here that down-regulation of mutant p53 activity, down-regulation of the Mcl-1 protein, and inhibition of aberrant Akt phosphorylation can provide promising therapeutic strategies for combination treatment with chemotherapy in a subset of patients with *FLT3*-ITD-positive AML. 

## Supplementary Materials

The following are available online at https://www.mdpi.com/1422-0067/20/5/1230/s1.
