# Supplementary material for: Cytarabine-Resistant FLT3-ITD Leukemia Cells are Associated with TP53 Mutation and Multiple Pathway Alterations—Possible Therapeutic Efficacy of Cabozantinib
Source: Int J Mol Sci. 2019 Mar 11;20(5):1230. doi: 10.3390/ijms20051230 (PMC6429333; doi:10.3390/ijms20051230)
Supplement: Supplementary file 1 [file ijms-20-01230-s001.pdf]

Supplementary

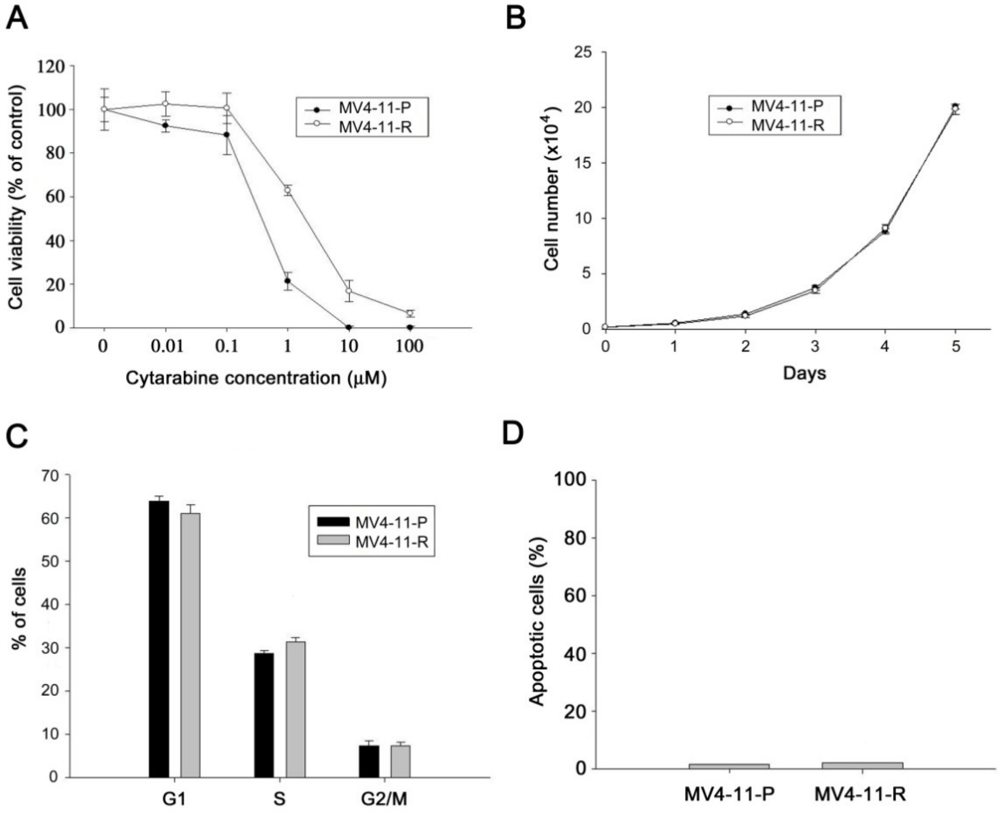

Figure S1. Cytotoxicity of cytarabine (A), growth curves (B), distribution of cell cycle phases (C), and apoptotic ratios (D) were assessed in MV4-11-P and MV4-11-R. For growth curves, cells were seeded on 6-well plates at 2500 cells/well. Viable cell numbers were counted after 24, 48, 72, 96, and 120 h by trypan blue exclusion method. For cell cycle and apoptosis analyses, cells were seeded on 6-well plates at 2.5 x 10^5 cells/well. After 24 h, cells were fixed, treated with RNase A, and stained with 5 μg/ml of propidium iodide for 10 min. Cell cycle distribution was analyzed using a Coulter EPICS XL-MCL flow cytometer (Beckman Coulter, Inc., Brea, CA, USA) with EXPO32 and MultiCycle software. Cells in the sub-G1 fraction was considered apoptotic cells.

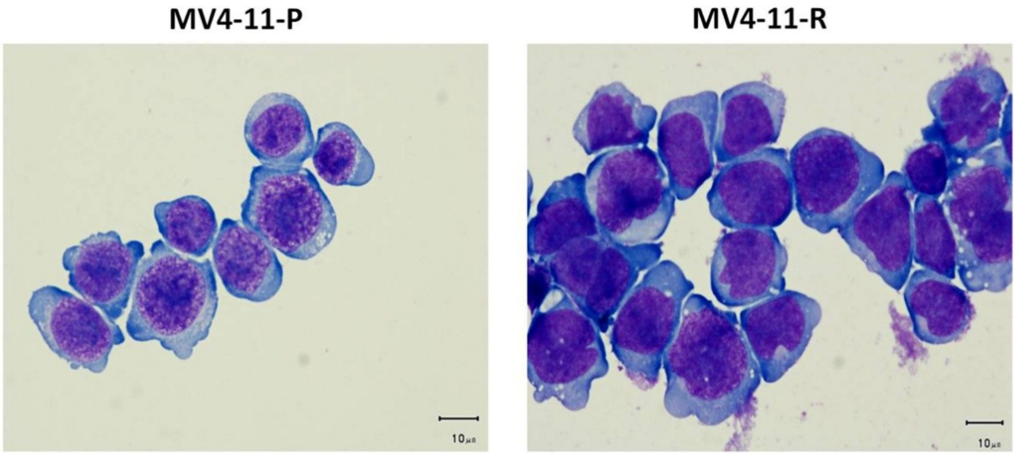

Figure S2. Morphological features of MV4-11-P and MV4-11-R were observed using Liu's staining. Scale bars = 10 μM.

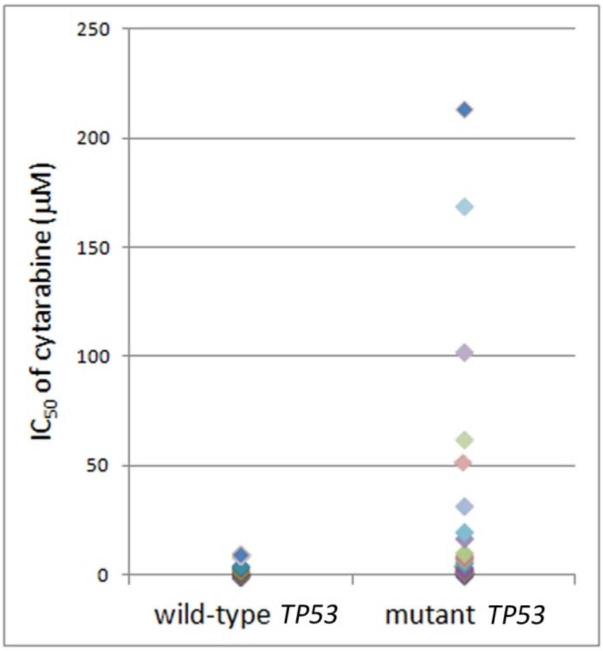

	No. of cell lines	average	SD
wild-type *TP53*	12	1.91	2.82
mutant *TP53*	36	21.07	44.03

Figure S3. Comparison of IC_{50} values of cytarabine between cancer cell lines harboring wild-type and mutant *TP53*. The NCI-60 cell lines with IC_{50} values of cytarabine available from the online database CancerDR (http://crdd.osdd.net/raghava/cancerdr/index.html) were included for comparison. (SD: standard deviation; $p = 0.0077$)

Table S1. Expression levels of cell surface markers in MV4-11-P and MV4-11-R.

	MV4-11-P	MV4-11-R
Marker	Percentage	Percentage
CD 45	98.4%	96.7%
CD 1b	0.2% - 0.7%	0.1% - 0.7%
CD 32	0.1% - 0.5%	0.1% - 0.6%
CD 34	0.6% - 11.9%	0.3% - 18.6%
CD 117	0.6% - 1.8%	0.1% - 2.6%
HLA-DR	12.7%	2.4%
CD 3	0.3%	0.3%
CD 20	0.1%	0.3%
CD 19	0.2%	0.2%
CD 5	0.1%	0.3%
CD 7	0.1%	0.2%
CD 33	0.1%	0.2%
CD 11b	0.1%	0.4%
CD 56	22.8%	37.2%
CD 38	0.4%	0.8%
GPA	0.2%	0.1%
CD 41	0.2%	0.3%
CD 14	0.4%	0.2%
CD 2	0.5%	0.4%
CD 13	2.3%	1.3%
CD 16	5.7%	8.8%
IgM	0.6%	0.4%
CD 15	100.0%	100.0%

Table S2. List of NCI-60 cell lines, p53 mutant protein variants, pathogenicity of p53 mutants, and cytarabine IC_{50} data.

Cell line	Cancer	p53_variant[a]	Pathogenicity[b]	Cytarabine IC_{50} (μM)[c]
MOLT-4	Leukemia/lymphoma	p.R306*	Pathogenic	0.09
OVCAR-8	Ovarian carcinoma	p.0?	-	0.21
SW620[d]	Colorectal carcinoma	p.P309S<br>p.R273H	Possibly pathogenic<br>Pathogenic	0.35
HOP-92	Lung (NSCLC)	p.R175L	Possibly pathogenic	0.36
BT-549	Breast carcinoma	p.R249S	Pathogenic	0.53
SN12C	Renal cell carcinoma	p.E336*	Pathogenic	0.62
786-0[d]	Renal cell carcinoma	p.0?<br>p.P278A	-<br>Likely pathogenic	0.67
RXF393	Renal cell carcinoma	p.R175H	Pathogenic	0.70
SF-539	Glioblastoma	p.R342fs*3	Likely pathogenic	0.72
SK-MEL-28	Melanoma	p.L145R	Likely pathogenic	0.80
Hs-578-T	Breast carcinoma	p.V157F	Pathogenic	0.84
U251	Glioblastoma	p.R273H	Pathogenic	1.13
NCI-H23	Lung (NSCLC)	p.M246I	Possibly pathogenic	1.20
SF-268	Glioblastoma	p.R273H	Pathogenic	1.36
TK10	Renal cell carcinoma	p.L264R	Possibly pathogenic	1.73
IGROV-1[d]	Ovarian carcinoma	p.P90fs*59<br>p.Y126C	Likely pathogenic<br>Pathogenic	1.79
HL-60	Leukemia/lymphoma	p.0	-	2.44
NCI-H226	Lung (NSCLC)	p.R158L	Pathogenic	2.51
HCC-2998	Colorectal carcinoma	p.R213*	Pathogenic	2.57
HT-29	Colorectal carcinoma	p.R273H	Pathogenic	3.51
SK-MEL-2	Melanoma	p.G245S	Pathogenic	4.01
SF-295	Glioblastoma	p.R248Q	Pathogenic	4.02
OVCAR-4	Ovarian carcinoma	p.L130V	Likely pathogenic	6.04
HCT-15	Colorectal carcinoma	p.0?<br>p.S241F	-<br>Pathogenic	8.07
MDA-MB-231	Breast carcinoma	p.R280K	Pathogenic	8.77

SNB-75	Glioblastoma	p.E258K	Pathogenic	9.30
KM12[e]	Colorectal carcinoma	p.H179R<br>p.V73fs*50	Pathogenic<br>-	10.21
HOP-62	Lung (NSCLC)	p.0?	-	16.46
OVCAR-3	Ovarian carcinoma	p.R248Q	Pathogenic	20.13
CCRF-CEM	Leukemia/lymphoma	p.R175H<br>p.R248Q	Pathogenic<br>Pathogenic	31.78
T47D	Breast carcinoma	p.L194F	Likely pathogenic	31.85
DU-145	Prostate carcinoma	p.P223L<br>p.V274F	Possibly Pathogenic<br>Pathogenic	51.50
RPMI-8226	Leukemia/lymphoma	p.E285K	Pathogenic	61.72
K-562	Leukemia/lymphoma	p.Q136fs*13	Likely pathogenic	102.03
SK-OV-3	Ovarian carcinoma	p.P90fs*33	Likely pathogenic	169.00
EKVX	Lung (NSCLC)	p.E204*	Likely pathogenic	213.55
A-549	Lung (NSCLC)	Wild type	-	0.05
CAKI-1	Renal cell carcinoma	Wild type	-	0.12
OVCAR-5	Ovarian carcinoma	Wild type	-	0.22
HCT-116	Colorectal carcinoma	Wild type	-	0.25
LOXIMVI	Melanoma	Wild type	-	0.29
ACHN	Renal cell carcinoma	Wild type	-	0.37
UACC-62	Melanoma	Wild type	-	0.67
UO-31	Renal cell carcinoma	Wild type	-	1.25
MCF-7	Breast carcinoma	Wild type	-	2.00
A498	Renal cell carcinoma	Wild type	-	3.71
UACC-257	Melanoma	Wild type	-	4.44
NCI-H460	Lung (NSCLC)	Wild type	-	9.57

[a]Leroy, B.; Girard, L.; Hollestelle, A.; Minna, J. D.; Gazdar, A. F.; Soussi, T., Analysis of TP53 mutation status in human cancer cell lines: a reassessment. Human mutation 2014, 35, (6), 756-65.

[b]The TP53 Web Site. Available online: https://p53.fr/ (accessed on 2018/12/22).

[c]CancerDR: Cancer Drug Resistance Database. Available online: http://crdd.osdd.net/raghava/cancerdr/ (accessed on 2018/12/22).

[d]Cell line with two *TP53* mutations.

[e]Cell line with three *TP53* mutations.

Table S3. Association of *TP53* mutation with resistance to anti-cancer drugs assessed by data of pancancer cell lines from Genomics of Drug Sensitivity in Cancer. Available online: https://www.cancerrxgene.org/.

**Drug**	**Drug target**	**Effect size**	**P-value**	**FDR%**	**No. of screened cell lines**	**No. of *TP53* mutated cell lines**
cytarabine	antimetabolite	0.284	0.0321	73.9	876	552
CI-1040	MEK1, MEK2	0.255	0.00882	20.3	869	549
cabozantinib	VEGFR, MET, RET, KIT, FLT1, FLT3, FLT4, TIE2, AXL	0.117	0.618	79.7	975	607
sorafenib	PDGFR, KIT, VEGFR, RAF	0.131	0.819	87.7	430	249
MK-2206	AKT1, AKT2	-0.0641	0.0606	97.6	852	536
